# Effect of Croatian propolis on diabetic nephropathy and liver toxicity in mice

**DOI:** 10.1186/1472-6882-12-117

**Published:** 2012-08-06

**Authors:** Nada Oršolić, Damir Sirovina, Marijana Zovko Končić, Gordana Lacković, Gordana Gregorović

**Affiliations:** 1 Department of Animal Physiology, Faculty of Science, University of Zagreb, Rooseveltov trg 6, Zagreb HR-10000, Croatia; 2 Faculty of Pharmacy and Biochemistry, University of Zagreb, A. Kovačića 1, Zagreb 10000, Croatia; 3 Department of Zoology, Faculty of Science, University of Zagreb, Rooseveltov trg 6, Zagreb 10000, Croatia

**Keywords:** Alloxan, Diabetes, Mice, Propolis, Liver, Kidney

## Abstract

**Background:**

In the present study, we examined the antioxidant effect of water soluble derivative of propolis (WSDP) and ethanolic (EEP) extract of propolis on renal and liver function in alloxan-induced diabetic mice. In addition, we examined whether different extract of propolis could prevent diabetic nephropathy and liver toxicity by inhibiting lipid peroxidation *in vivo*.

**Methods:**

Diabetes was induced in Swiss albino mice with a single intravenous injection of alloxan (75 mg kg^-1^). Two days after alloxan injection, propolis preparations (50 mg kg^-1^ per day) were given intraperitoneally for 7 days in diabetic mice. Survival analysis and body weights as well as hematological and biochemical parameters were measured. The renal and liver oxidative stress marker malonaldehyde levels and histopathological changes were monitored in the liver and kidney of treated and control mice.

**Results:**

Administration of propolis to diabetic mice resulted in a significant increase of body weight, haematological and immunological parameters of blood as well as 100% survival of diabetic mice. Alloxan-injected mice showed a marked increase in oxidative stress in liver and kidney homogenate, as determined by lipid peroxidation. Histopathological observation of the liver sections of alloxan-induced diabetic mice showed several lesions including cellular vacuolization, cytoplasmic eosinophilia and lymphocyte infiltrations, but with individual variability.Treatment of diabetic mice with propolis extracts results in decreased number of vacuolized cells and degree of vacuolization; propolis treatment improve the impairment of fatty acid metabolism in diabetes. Renal histology showed corpuscular, tubular and interstitial changes in alloxan-induced diabetic mice. Test components did not improve renal histopathology in diabetic mice.

**Conclusions:**

Propolis preparations are able to attenuate diabetic hepatorenal damage, probably through its anti-oxidative action and its detoxification proccess as well as the potential to minimize the deleterious effects of free radicals on tissue. The protective role of propolis against the ROS induced damages in diabetic mice gives a hope that they may have similar protective action in humans.

## Background

Diabetes mellitus is possibly the World’s fastest growing metabolic disorder, and as the knowledge of the heterogeneity of this disorder increases, so does the need for more appropriate therapy [1]. Diabetes mellitus is a pathologic condition, resulting in severe metabolic imbalances and non-physiologic changes in many tissues, where oxidative stress plays an important role in the aetiology [1,2]. Diabetes is associated with the generation of reactive oxygen species (ROS), which cause oxidative damage, particularly to heart, kidney, eyes, nerves, liver, small and large blood vessels, immunological and gastrointestinal system [1,3].

Diabetic nephropathy (DN) is one of the important microvascular complications of diabetes mellitus. Recent studies indicate that reactive oxygen species (ROS) play a key intermediate role in the pathophysiology of diabetic nephropathy [4]. Hyperglycaemia, the main determinant of the initiation and progression of diabetic nephropathy, not only generates more reactive oxygen metabolites, but also attenuates anti-oxidative mechanisms through nonenzymatic glycosylation of anti-oxidant enzymes [1]. The mechanism by which hyperglycaemia causes free radical generation and, thus, causes oxidative stress is complex. High glucose concentration directly increases hydrogen peroxide production by murine mesangial cells and lipid peroxidation of glomeruli and glomerular mesangial cells [5]. Hyperglycaemia promotes glycosylation of circulating and cellular protein and may initiate a series of auto-oxidative reactions that culminate in the formation and accumulation of advanced glycosylation end-products (AGE) in tissue proteins [6,7]. The AGE has oxidizing potential and can promote tissue damage by free radicals. In addition, increased lipid peroxidation impairs membrane functions by decreasing membrane fluidity and changing the activity of membrane-bound enzymes and receptors. Its products (lipid radicals and lipid peroxides) are harmful to the cells in the body and associated with atherosclerosis and damage to brain, kidney, liver and other tissue [1,8]. In addition, diabetes and hyperglycemia can be sources of DNA damage via the oxidation of DNA bases and sugar-phosphate binding sites [9]. The occurrence of these alterations can result in mutagenic effects and/or DNA replication arrest, and could be associated with risks for developing cancer in diabetes mellitus patients [10,11].

Propolis is a complex resinous material collected by honeybees from buds and exudates of certain plant sources neighbouring its hives. Propolis consisting of sap, bark and bee excreta accumulates in bee hives. Complexity and variety of the chemical composition of propolis has been reported in the literature. From different botanical and geographical origins of world, more than 300 compounds including volatile organic compounds, flavonoid aglycones, phenolic acids and their esters, phenolic aldehydes, alcohols and ketones, sesquiterpenes, quinones, coumarins, steroids, aminoacids were reported to have been isolated from propolis [12-14]. The main types of flavonoids are galangin, chrisin, pinocebrin and caffeic acid phenethyl ester [13-15].

Propolis possesses a broad spectrum of biological activities and has a historical utilization in folk medicine. Thus, it is extensively being used in health food, pharmaceutical preparations [16] and beverages with the aim of maintaining or improving human health [1]. It was reported that propolis enhances immune system activities [17-20], oxygen radical scavenging [21], antimicrobial [22-24] and antitumor activities [18,25,26].

Flavonoids, present in propolis, are plant phenolic compounds with strong antioxidant properties. Propolis, a potent anti-oxidant, scavenges free radicals directly [27-29], inhibits xanthine oxidase [30], inhibits lipid peroxidation [31] and alters anti-oxidant defence pathway *in vivo* and *in vitro*[32].

Recently, it has been reported that propolis inhibits oxidative damage in various tissues of alloxan- or streptozotocin-induced diabetic animals [16,33]. And while the people spontaneously develop diabetes, in animals it can be induced by partial pancreatectomy or by the administration of diabetogenic drugs such as alloxan, streptozotocin, ditizona and anti-inzulin serum. Alloxan can induce several processes: oxidation of –SH groups, inhibition of glucokinase, generation of free radicals and disturbances in calcium homeostasis [9]. Such data support the use of alloxan-induced diabetes as a model for the oxidative stress status experienced by diabetic patients.

The present study was designed to investigate the effect of different extracts of poplar-type propolis (water and ethanolic solution) on lipid peroxidation, renal and liver functions, and possibilities of natural anti-oxidants, such as propolis, to protect or attenuate the damage of the kidney and liver in alloxan-induced diabetic mice.

## Methods

### Animals

Present study was approved by the ethical committee (Faculty of Science, University of Zagreb*,* Croatia). Male and female Swiss albino inbred mice 2 to 3 months old, weighing 20 to 25 g, obtained from Department of Animal Physiology, Faculty of Science, University of Zagreb, were used in this study. The animals were kept in individual cages during the experiment and at 12 hours of light per day. They were fed a standard laboratory diet (4 RF 21, Mucedola, Settimo Milanese, Italy) and tap water ad libitum. Maintenance and care of all experimental animals were carried out according to the guidelines in force in Republic of Croatia (Law on the Welfare of Animals, N.N. #19, 1999) and carried out in compliance with the Guide for the Care and Use of Laboratory Animals, DHHS Publ. # (NIH) 86–123.

### Water-soluble derivative of propolis (WSDP)

Row Croatian propolis was collected by scraping it off from hive frames. The collected propolis samples were kept desiccated in the dark until analysis at room temperature. Water-soluble derivative of propolis (WSDP) was prepared by the method described in our previous paper [17]. Briefly, Croatian propolis from beehives kept at the outskirts of Zagreb was extracted with 96% ethanol, which was filtered and evaporated to dryness in vacuum evaporator. The resultant resinous product was added to a stirred solution of 8% L-lysine (Sigma Chemie, Deisenhofen, Germany) and freeze-dried to yield the WSDP, a yellow-brown powder.

The chemical profile of propolis from the northern hemisphere, often named as “poplar-type” propolis can be characterized by the three analytical parameters: total flavonol and flavone content, total flavanone and dihydroflavonol content, and total polyphenolics content. According to Popova et al. [34], spectrophotometric procedures for quantification of the three main groups of bioactive substances in propolis could be used for quality assessment of different propolis samples, and results of those analyses correlate with biological activity, especially in the “poplar-type” of propolis. The spectrophotometric assay based on the formation of aluminium chloride complex was applied for quantification of total flavones/flavonols and expressed as quercetin equivalent. For the quantification of flavanones and dihydroflavonols propolis, we used 2,4-dinitrophenylhydrazine method [35]. Total polyphenolics content was measured by the Folin–Ciocalteu procedure [21]. Total phenol content was expressed as gallic acid equivalents (mg/g), total flavonoid contents as quercetin equivalents (mg/g), while total flavanones and dihydroflavonols content was expressed as naringenin equivalents (mg/g) from calibration curves recorded for the standards.

WSDP contained: flavones and flavonols 2.13%, flavanones and dihydroflavonols 9.06%, total flavonoids 11.19%, total polyphenols 70.48%.

### Ethanolic extract of propolis (EEP)

Ethanolic propolis extract (EEP) was prepared by the method described elsewhere [13,36]. Briefly, propolis (10 g) was crushed into small pieces in a mortar and mixed vigorously with 34.85 ml 80% (V/V) ethanol during 48 h at 37 ± 1°C. After extraction, the ethanolic extract of propolis was filtered through Whatman N_0_. 4 paper and than the extract was lyophilized. Spectrophotometric analysis has shown that EEP contained: flavones and flavonols 1.6%, flavanones and dihydroflavonols 38.60%, total flavonoids 40.20%, total polyphenols 84.40%.

### Antioxidant capacity of the extracts

#### β-Carotene−linoleic acid assay

The antioxidant activity of the extracts was evaluated using β-carotene−linoleic acid system according to Amarowicz et al. [37]. In short, 1 mL of β-carotene solution in chloroform (0.2 mg mL^-1^) was pipetted into a round-bottom flask. To the solution, 20 mg of linoleic acid and 200 mg of Tween 40 were added. After removing chloroform in a rotary evaporator, 50 mL of aerated distilled water was added to the oily residue. Aliquots (5 mL) of thus obtained emulsion were transferred to a series of tubes containing 2 mg of extract or 0.5 mg of BHA (positive control). Emulsion without antioxidant served as control. After addition of the emulsion to the tubes, they were placed in a water bath at 50°C for 2 h. During that period, the absorbance of each sample was measured at 470 nm at 15 min intervals, starting immediately after sample preparation (*t* = 0 min) until the end of the experiment (*t* = 120 min). The rate of β-carotene bleaching (*R*) for the extracts, BHA and water, was calculated according to first-order kinetics. The percent of antioxidant activity (ANT) was calculated as described in Al-Saikhan et al. [38], using the equation:

(1) $$\text{ANT}=({\text{R}}_{\text{control}}-{\text{R}}_{\text{sample}})/{\text{R}}_{\text{control}}\times 100$$

where *R*_control_ and *R*_sample_ are average bleaching rates of water control and antioxidant (plant extract or BHA), respectively.

### DPPH radical-scavenging activity

The scavenging effect for DPPH free radical was monitored as described in Zovko Končić at al. [39] with minor modification. Briefly, 1.0 mL of 0.16 mM DPPH methanolic solution was added to 1.0 mL of either methanolic solution of extract (sample) or methanol (control). The mixtures were vortexed and then left to stand at room temperature in the dark. After 30 min absorbance was read at 517 nm. Radical-scavenging activity (RSA) for DPPH free radical was calculated using the following equation:

(2) $$\;\text{RSA}=({\text{A}}_{\text{control}}-{\text{A}}_{\text{sample}})/{\text{A}}_{\text{control}}\times 100$$

where *A*_control_ is the absorbance of the methanol control and *A*_sample_ is the absorbance of the extract. Synthetic antioxidant, BHA, was used as positive control. DPPH radical-scavenging activity was calculated as the concentration that scavenges 50% of DPPH free radical and thus has RSA = 50% (EC_50_).

### The reducing power of the extracts

The reducing power of extracts was determined according to the method of Yen and Chen [40]. Briefly, extracts (0.2–1.0 mg) were dissolved in 1.0 mL of distilled water and mixed with 2.5 mL of 0.2 M phosphate buffer (pH 6.6) and 2.5 mL of 1% potassium ferricyanide. The mixtures were incubated at 50°C for 20 min. After incubation, 2.5 mL of a 10% trichloroacetic acid was added to the mixtures. Following that, samples were centrifuged for 10 min. Aliquots of 2.5 mL of the upper layer were combined with 2.5 mL of water and 0.5 mL of the 0.1% solution of ferric chloride. Absorbance of the reaction mixture was read spectrophotometrically at 700 nm. Ascorbic acid was used as positive control.

### Chelating activity (ChA)

The chelation of iron (II) ions was studied as described by Decker and Welch [41]. An aliquot of the extract in methanol (1.3 mL) was added to 100 μL of 2 mM FeCl_2_. After 5 min, the reaction was initiated by adding 200 µl of 5 mM ferrozine. Following 10 min incubation at room temperature, the absorbance at 562 nm was recorded. For preparation of control, 1.3 mL of methanol was used instead of extract solution. EDTA was used as a chelating standard. The Fe^2+^-chelating activity (ChA) was calculated using the equation below:

(3) $$\;\text{ANT}=({\text{A}}_{\text{control}}-{\text{A}}_{\text{sample}})/{\text{A}}_{\text{control}}\times 100$$

where *A*_control_ is the absorbance of the negative control (solution to which no extract was added) and *A*_sample_ is the absorbance of the extract solution. Chelating activity was expressed as ChEC_50_, the concentration that chelates 50% of Fe^2+^ ions and thus has ChA = 50%.

### Experimental design

Seventy mice were randomly divided into four groups, as follows:

*Group (i)*: control animals (healthy, nondiabetic animals); received 0.5 mL distilled water intraperitonealy (i.p.) per day by injection for 7 days;

*Group (ii)*: alloxan controls; injected i.v. with alloxan in a single dose of 75 mg kg^-1^ body weight; these served as the untreated diabetic group;

*Group (iii)*: received WSDP i.p. in a daily dose of 50 mg kg^-1^ for 7 days starting 2 days after alloxan injection; these served as the WSDP-treated diabetic group.

*Group (iv)*: received EEP i.p. in a daily dose of 50 mg kg^-1^ for 7 days starting 2 days after alloxan injection; these served as the EEP -treated diabetic group.

Five mice from each group were used on the 9^th^ day after alloxan injection. After desinfection of the external abdominal region, each animal was inoculated with 3 mL of saline solution and after gentle agitation of the abdominal wall, the solution containing peritoneal cells was removed for cellular evaluation. The following variables were analyzed: toxicity analysis, animal weight loss, hematological, biochemical parameters (total cholesterol and triglyceride), determination of lipid peroxidation of liver and kidney cells and their histopathological analysis.

The remaining animals, i.e., 8–11 animals of each group were used for the survival analysis (increased lifespan).

### Induction of experimental diabetes and determination of serum glucose level

Diabetes was induced in Swiss albino mice by a single intravenous injection of alloxan monohydrate (75 mg kg^-1^, i.v.) in total volume of 0.5 mL of freshly prepared saline solution. Blood glucose level was tested before alloxan injection and 48 h after treatment, to monitor the immediate diabetogenesis. After 48 h, the animals with blood glucose level above 11 mmol L^-1^ were selected for the study (diabetic mice) and then treated with WSDP or EEP. Blood glucose level was determined by test strips of blood glucose (Betachek Visual blood glucose test strips, Sydney, Australia).

### Effect of WSDP or EEP on body weight in alloxan induced diabetic mice

During the study period of 50 days, the body weights of the mice were recorded every 4 days using an electronic balance. From these data, the mean change in body weight was calculated. The maximum percentage of animal weight loss, an indicator of toxicity, was calculated for individual animals as:

(4) $$\%\text{ animal weight loss}=\left[\left(\text{Day}\;1\text{ weight}–\text{minimum weight on study}\right)/\text{Day}\;1\text{ weight}\right]\times 100$$

### Survival analysis

For the survival analysis Swiss albino mice were given test components i.p. at doses of 50 mg kg^-1^ for 7 days starting 2 days after the alloxan injection. The end point of the experiment was determined by the spontaneous death of animals. The results are expressed as percentage of mean survival time of the treated animals over the mean survival time of the control group with diabetes (treated vs. control, T/C%). The percentage of increased lifespan (ILS%) was calculated according the formula: $\text{ILS\%}=\left(\text{T}-\text{C}\right)/\text{C}\times 100$ where T represents mean survival time of treated animals and C represents mean survival time of the control group.

### Haematological analysis

The haematological analysis was performed on blood obtained from the tail vein of experimental and control mice on day 9 after alloxan injection. Blood was collected into EDTA tubes. The measurement of the leukocyte, erythrocytes, haemoglobin, hematocrit, MCV, MCH, MCHC and platelets was made in an automatic cell counter (Cell-Dyn® 3200, Abbott, USA).

### Serum samples and biochemical determinations

Animals were treated with test components, blood samples were collected and centrifuged at 2200 rpm for 10 minutes. Serum was used for the determination of total protein, glucose, urea, creatinine, bilirubin, alcaline phosphatase (ALP), aspartate and alanine aminotransferases (AST and ALT) and lactic dehydrogenase (LDH). Biochemical parameters were made using serum samples from both control and experimental groups in an automatic cell counter. Serum triglycerides and total cholesterol were determined by enzymatic methods according to the commercial kit’s instructions (Thermo Electron, Australia). The total concentration of triglycerides or total cholesterol was estimated by measuring the absorbance of sample and standard by spectrophotometer (Shimadzu, UV-160) at a wavelength of 500 nm.

### Prepations of tissue homogenate and protein estimation

Samples of liver and kidneys (100 mg mL^-1^ buffer) were homogenized in 50 mM phosphate buffer (pH 7.0), and then centrifuged at 10,000 rpm for 15 min; the supernatant thus obtained was used for biochemical analysis. The protein concentration in each fraction was determined by the standard laboratory procedure [42], at NanoDrop 1000 Spectrophotometer V3.7. using crystalline bovine serum albumin as standard.

### Determination of lipid peroxidation

The extent of lipid peroxidation was determined by the method of Ohkawa et al. [43]. To a tube containing 0.2 mL of 8.1% sodium dodecyl sulfate, 1.5 mL of 20% acetic acid (pH 3.5), and 1.5 mL of 0.81% thiobarbituric acid aqueous solution were added. To this reaction mixture, 0.2 mL of each tissue homogenate was added. The mixture was then heated in boiling water for 60 min. After cooling to room temperature, 5 mL of butanol:pyridine (15:1, v/v) solutions were added. The mixture was then centrifuged at 5000 rpm for 15 min. The upper organic layer was separated, and the intensity of the resulting pink color was read at 532 nm. Tetramethoxypropane was used as an external standard. The level of lipid peroxides was expressed as nmoles of malondialdehyde (MDA) formed/mg protein.

### Histopathological analysis

For the histopathological changes, liver and kidney tissues from diabetic control mice treated with physiological and ethanolic solution and diabetic mice treated with WSDP and EEP were fixed in 10% neutral buffered formalin for 24 hours, dehydrated in a graded alcohol series and after chloroform treatment embedded in paraplast. Deparaplasted 5–6 μm thick sections were stained with hematoxylin and eosin (HE) following standard protocol. Stained slides were examined under a light microscope (Nikon Eclipse E600) at 100, 200, 400 and 1000x magnification. Liver sections were examined for vacuolization, lymphocyte infiltrations, necrosis and apoptosis. The percentage of apoptotic cells was determined by counting 200 cells in randomly selected microscopical fields of vision.

Kidney sections were examined for lymphocyte infiltrations, reduction of Bowman’s spaces and changes in renal tubules.

Photomicrographs were taken by digital camera (Nikon DMX1200) and Imaging Software Lucia G 4.80 (Laboratory Imaging Ltd., Prague, Czechoslovakia).

### Statistical analyses

The experiments were performed in triplicate. The results were expressed as mean ± SD. Statistical comparisons were made using one-way ANOVA, followed by Dunnett’s post-hoc test for multiple comparisons with the control and Student’s *t*-test for comparison between samples. Statistical analyses were performed using the JMP V6 from SAS software (SAS Institute, Cary, NC, USA). A value of *P* < 0.05 was considered to indicate statistical significance.

## Results

### Effect of alloxan on blood glucose level

Alloxan at dose of 75 mg kg^-1^ successfully causes diabetes in mice. Blood glucose level was strongly elevated on the second day after treatment and the average levels of blood glucose in each group of mice ranging between 20–30 mmol L^-1^ (data not shown).

### Effect of WSDP or EEP on body weight and survival of alloxan-induced diabetic mice

**Figure 1:**
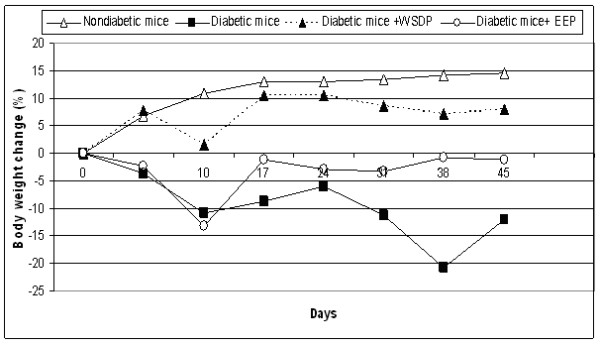
**Effect of WSDP or EEP on body weight of alloxan-induced diabetic mice.** Control group of nondiabetic mice received 0.5 mL physiological solution intraperitonealy (*ip*) for 7 consecutive days starting starting 2 days after aloxan injection.Diabetic mice; injected *iv* with alloxan in a single dose of 75 mg kg^-1^ body weight and served as untreated diabetic group.Untreated diabetic mice received 0.5 mL physiological solution intraperitonealy (*ip*) or 0.5% ethanolic solution for 7 consecutive days starting starting 2 days after aloxan injection. Diabetic mice treated with water-soluble derivative of propolis (WSDP) or ethanoloc extract of propolis (EEP) *ip* in a daily dose of 50 mg kg^-1^ for 7 days starting 2 days after alloxan injection.

Effect of WSDP or EEP on body weight of mice with alloxan–induced diabetes is shown in Figure 1 and Table 1. Body weight was rapidly reduced in animals treated with alloxan alone; the fall was the largest between 3 and 10 days, and then body weight started to recover easily, especially in diabetic animals treated with WSDP, which have almost reached a mass of healthy (nondiabetic) animals. During the experiments, 3 or 4 animals died from the untreated group of mice with diabetes, while animals treated with test components are all survivors as shown in Table 1. Increased life span (ILS) was observed in treated diabetic mice with both WSDP and EEP preparations for 30.43% and 20.67%, respectively, in relation to control diabetic mice.

**Table 1:** Survival of untreated diabetic mice and diabetic mice treated with WSDP or EEP

**Experimental group**	**Range of survival (days)**	**Median ± SD**	**ILS%**^**c**^	**T/C%**^**c**^	**Long-term survivors (LTS)**^**f**^
Diabetic mice^a^	15-45	38.5 ± 13.12	-	-	3
Diabetic mice + (0.5% EtOH)	22-45	42 ± 9.68	-	-	4
Diabetic mice + WSDP^b^	45	45*	30.43	130.43	8
Diabetic mice + EEP^b^	45	45*	20,67	120,67	8

^a^Diabetic mice; injected *iv* with alloxan in a single dose of 75 mg kg^-1^ body weight and served as untreated diabetic group. Untreated diabetic mice received 0.5 mL physiological solution or 0.5% ethanolic solution intraperitonealy (*ip*) for 7 consecutive days starting starting 2 days after aloxan injection.

^b^Diabetic mice treated with WSDP or EEP *ip* in a daily dose of 50 mg kg^-1^ for 7 days starting 2 days after alloxan injection.

^c^ILS% (increased life span%) = (T-C)/Cx100. T, mean survival time of treated diabetic group; C, mean survival time of untreated diabetic group.

^d^T/C treated diabetic animals *versus* untreated diabetic animals.

^e^LTS- Long-term survivors; mice surviving more than 45 days after alloxan treatment.

Results are expressed as the means of each group (n = 8) of and are representative of two independent experiments.

*Statistically significant difference between WSDP or EEP treated diabetic mice and untreated diabetic animals (*P <0.05; Log-Rank test).

### Effect of WSDP or EEP on haematological and biochemical parameters

Tables 2 and 3 show haematological and biochemical parameters in normal (nondiabetic) animals and experimental animals in each group. The level of the total number of red blood cells, haemoglobin and hematocrit level were significantly elevated in diabetic mice treated with WSDP or EEP preparations (RBC, and HGB, *P* <0.05; HCT, *P* <0.01 or *P <*0.05) compared to untreated diabetic mice. Significantly reduced values of the platelet number are observed in the group of animals treated with both propolis preparations (WSDP, EEP) compared with diabetic mice without treatment (*P* <0.05). Other haematological parameters show no statistically significant differences (Table 2).

**Table 2:** Haematological parameters of nondiabetic mice, untreated diabetic mice and diabetic mice treated with WSDP or EEP

	**Nondiabetic mice**^**a**^	**Diabetic mice**^**b**^	**Diabetic mice + (0.5% EtOH)**	**Diabetic mice + EEP**^**c**^	**Diabetic mice + WSDP**^**c**^
WBC (×10^9^ L^-1^)	4.85 ± 1.00	4.96 ± 0.81	4.41 ± 1.31	4.59 ± 3.18	3.3 ± 1.42
Mononuclear/%	57.06 ± 12.08	63.16 ± 6.24	51.73 ± 12.62	56 ± 19.90	53.38 ± 9.63
Polymorphonuclear/%	42.94 ± 12.00	36.84 ± 6.24	48.27 ± 12.62	44 ± 19.90	46.62 ± 9.63
RBC (x 10^12^ L^-1^)	9.13 ± 0.32	9.18 ± 0.33	9.435 ± 0.28	9.87 ± 0.46 ^*Δ^	9.755 ± 0.14* ^**Δ**^
HGB (g L^-1^)	135.55 ± 5.35	132.05 ±5.25	136.2 ± 3.67	139.85 ± 5.56	139.45 ± 1.75*
HCT (L L^-1^)	0.444 ± 0.02	0.44 ± 0.01	0.452 ± 0.01	0.47 ± 0.02*	0.4715 ± 0.00**
MCV (fL)	97.2 ± 1.91	96.2 ± 2.33	95.8 ± 1.50	95.4 ± 1.59	96.75 ± 1.84
MCH (pg)	29.7 ± 0.26	28.8 ± 0.69	28.9 ± 0.41	28.3 ± 0.50	28.6 ± 0.43
MCHC (g L^-1^)	616.7 ± 6.43	598.5 ± 5.26	603 ± 7.74	593.5 ± 1.91	591.5 ± 4.43
RDW (%CV)	24 ± 3.00	23.1 ± 1.16	23.6 ± 1.78	22.35 ± 1.30	22.35 ± 1.11
PLT (x 10^9^ L^-1^)	1434.5 ± 373.75	1333.5 ± 330.52	1521.5 ± 251.01	805 ± 344.36^* **Δ**^	986 ± 151.85
MPV (fL)	9.76 ± 0.67	10.19 ± 0.24	9.92 ± 0.63	10.76 ± 1.05	9.97 ± 0.57

^a^Control group of nondiabetic mice received 0.5 mL physiological solution intraperitonealy (*ip*) for 7 consecutive days starting starting 2 days after alloxan injection.

^b^Diabetic mice; injected *iv* with alloxan in a single dose of 75 mg kg^-1^ body weight and served as untreated diabetic group. Untreated diabetic mice received 0.5 mL physiological solution or 0.5% ethanolic solution intraperitonealy (*ip*) for 7 consecutive days starting starting 2 days after aloxan injection.

^c^Diabetic mice treated with WSDP or EEP *ip* in a daily dose of 50 mg kg^-1^ for 7 days starting 2 days after alloxan injection.

*Statistically significant difference between WSDP or EEP treated diabetic mice and untreated diabetic animals (*P <0.05; ** *P* <0.01; Student *t*-test).

^**Δ**^ Statistically significant difference between nondiabetic animals and WSDP or EEP treated diabetic mice (^**Δ**^*P* <0.05; ^**ΔΔ**^*P* <0.01; Student *t*-test).

**Table 3:** Biochemical parameters of nondiabetic mice, untreated diabetic mice and diabetic mice treated with WSDP or EEP

	**Nondiabetic mice**^**a**^	**Diabetic mice**^**b**^	**Diabetic mice + (0.5% EtOH)**	**Diabetic mice + EEP**^**c**^	**Diabetic mice + WSDP**^**c**^
ALP (U L^-1^)	53.75 ± 7.50	83.33 ± 16.07^#^	95 ± 10.80^**##**^	71.25 ± 20.15	65 ± 10.80
AST (U L^-1^)	146.25 ± 13.50	186.25 ± 31.72	183.75 ± 41.70	167.5 ± 29.58	161.25 ± 29.54
ALT (U L^-1^)	57.5 ± 9.36	81.25 ± 9.46	90 ± 15.35	66.25 ± 11.09*	60 ± 10.80*
LD (g L^-1^)	1881 ± 298.48	2246.25 ± 157.92^#^	2487.5 ± 253.42^#^	995 ± 358.86^*** **ΔΔΔ**^	586.25 ± 90.68**^ΔΔ^
TP (g L^-1^)	48.5 ± 4.38	44.5 ± 1.96	48.75 ± 4.92	40.75 ± 4.78	40.875 ± 1.93 ^**Δ**^
GLU (mmol L^-1^)	9.89 ± 0.64	30.52 ± 5.34^**##**^	25.575 ± 4.55^**#**^	32.57 ± 7.47 ^**ΔΔΔ**^	47.75 ± 6.35 ^**ΔΔ**^
UREA (mmol L^-1^)	7.5 ± 1.22	9.125 ± 0.42	9.88 ± 1.89	13.625 ± 3.45 ^**Δ**^	7.88 ± 2.43
Cholesterol (mmol L^-1^)	3.52 ± 0.26	3.20 ± 1.00	3.20 ± 0.80	4.14 ± 2.36	3.55 ± 0.72
Triglycerides (mmol L^-1^)	2.72 ± 0.16	3.00 ± 0.90	3.37 ± 1.11	2.20 ± 0.97	2.56 ± 0.92

^a^Control group of nondiabetic mice received 0.5 mL physiological solution intraperitonealy (*ip*) for 7 consecutive days starting starting 2 days after aloxan injection.

^b^Diabetic mice; injected *iv* with alloxan in a single dose of 75 mg kg^-1^ body weight and served as untreated diabetic group.Untreated diabetic mice received 0.5 mL physiological solution or 0.5% ethanolic solution intraperitonealy (*ip*) for 7 consecutive days starting starting 2 days after aloxan injection.

^c^Diabetic mice treated with WSDP or EEP *ip* in a daily dose of 50 mg kg^-1^ for 7 days starting 2 days after alloxan injection.

*Statistically significant difference between WSDP or EEP treated diabetic mice and untreated diabetic animals (*P <0.05; P** < 0.01; Student *t*-test).

^**#**^ Statistically significant difference between untreated diabetic mice and nondiabetic animals (^**##**^*P* <0.01; Student *t*-test).

^**Δ**^ Statistically significant difference between nondiabetic animals and WSDP or EEP treated diabetic mice (^**Δ**^*P* <0.05; ^**ΔΔ**^*P* <0.01; ^**ΔΔΔ**^*P* <0.001; Student *t*-test).

As shown in Table 3 alloxan elevated values of ALP and LDH; a statistically significant difference exists between diabetic mice (*P* <0.05 or *P* <0.01) and nondiabetic animals. In diabetic mice treated with WSDP or EEP there was a reduction in levels of ALT (*P* <0.05), ALP, and LDH (*P* <0.01 or *P <*0.001), in relation to animals with diabetes without treatment. Statistically significant increase in levels of urea was noticed in diabetic groups treated with EEP (*P* <0.05) compared to healthy (nondiabetic) animals. Trigliceride levels were lower in propolis-treated diabetic mice than in untreated diabetic mice but there was no statistically significant (Table 3).

### Histopatological analysis

#### Liver

**Figure 2:**
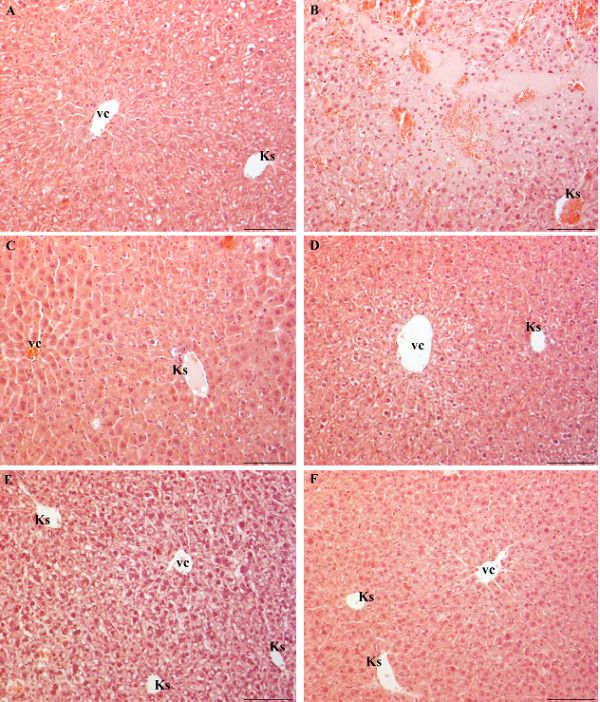
**Liver of diabetic mice stained with HE.** (A-B) Diabetic mice treated with physiological solution. (**A**) Hepatocytes around Kiernan’s spaces (Ks) are more vacuolized then cells around central vein (vc). (**B**) Focal hemorrhagic necrosis. (**C**) Diabetic mice treated with WSDP show less vacuolized hepatocytes. Dilatated sinusoids and swollen hepatocytes without vacuoles can be seen around central vein (vc). Cells around Kiernan’s space (Ks) are not swollen, some are weakly vacuolized. (D-E) Diabetic mice treated with ethanolic solution (0.5% EtOH). (**D**) Hepatocytes around central vein (vc) are more intensively vacuolized then cells around Kiernan’s space (Ks). (**E**) Increased number and level of vacuolized hepatocytes in whole lobule. (**F**) Diabetic mice treated with EEP. Hepatocytes are less vacuolized compared to diabetic mice. Bar = 20 μm.

In the liver of control diabetic mice, treated with a physiological solution, the majority of hepatocytes contained empty vacuole-like spaces. Hepatocytes around Kiernan’s spaces were more intensively vacuolized than the cells around central veins (Figure 2A). In contrast, in one sample, hepatocytes around central veins were more intensively vacuolized than the hepatocytes around Kiernan’s spaces. In all samples, small lymphocyte infiltrations were observed in parenchyma and beside vessels in some Kiernan’s spaces. Focal hemorrhagic necrosis was also found (Figure 2B).

In diabetic mice treated with WSDP, half of the samples were similar to control, however the hepatocytes were less vacuolized, but not significantly. In the other half of the samples histological image was quite different. Sinusoids were dilatated and hepatocytes around central veins were swollen but not vacuolized (Figure 2C). In the area around Kiernan’s spaces, small amount of weakly vacuolized hepatocytes were observed (Figure 2C). The rest of the hepatocytes were not vacuolized nor swollen. In these samples smaller lymphocyte infiltrations were found only in parenchyma. Focal hemorrhagic necrosis was not found. Generally, the liver of diabetic mice treated with WSDP showed lower amount of vacuolized hepatocytes with less vacuolization.

In the liver of control diabetic mice treated with an ethanolic solution, in half of the samples, hepatocytes around central veins were more intensively vacuolized, compared to the hepatocytes around Kiernan’s spaces (Figure 2D). Smaller lymphocyte infiltrations were found beside vessels in some Kiernan’s spaces.

In the other half of the samples, almost all hepatocytes were significantly vacuolized, with no difference in the level of cell vacuolization between the central and peripheral part of the lobules (Figure 2E). In these samples, lymphocyte infiltrations were not found.

In diabetic mice treated with EEP, hepatocytes around central veins were more intensively vacuolized compared to the hepatocytes around Kiernan’s spaces, but in the majority of the samples they were less vacuolized according to the control (Figure 2F). In these samples, smaller lymphocyte infiltrations were found beside vessels in some Kiernan’s spaces, as well as in parenchyma. Focal hemorrhagic necrosis was also found.

**Figure 3:**
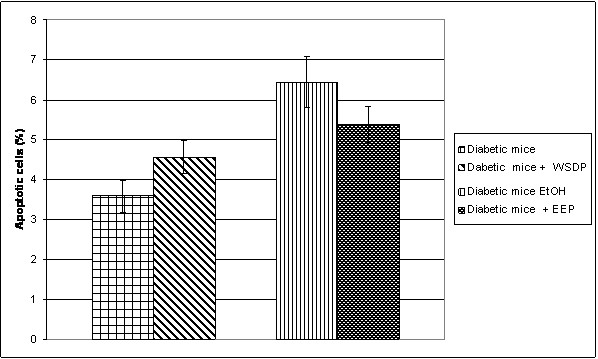
**Number of apoptotic cells in the liver of diabetic mice treated with physiological and ethanolic solution (0.5% EtOH) and diabetic mice treated with WSDP and EEP.** Data are mean ± SD and represent percentages of apoptotic cells in total of 200 cells.

Generally, hepatocytes in diabetic mice treated with EEP were less vacuolized compared to untreated diabetic mice, but not significantly.

n the liver of all diabetic mice exposed to physiological and ethanolic solution separately as well as in combination with propolis, necrotic and apoptotic cells were found. The quantitative analysis of apoptotic cells is shown in Figure 3. The results showed that the liver of diabetic mice after treatment with EEP had 20% less apoptotic cells compared to adequate control. WSDP had no effect in preventing apoptosis.

### Kidney

**Figure 4:**
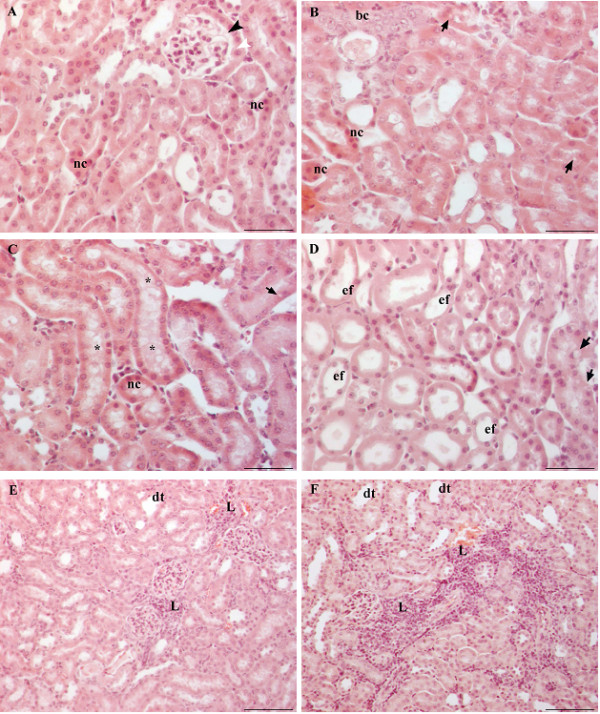
**Kidney of diabetic mice stained with HE.** (A-E) Diabetic mice treated with physiological solution. (**A**) The renal corpuscle with narrow Bowman’s space (black arrowhead) and columnar cells in the parietal layer of Bowman’s capsule (white arrowhead). Tubular changes involve: (A-D) presence of necrotic cells (nc)**,** (**B**) presence of basophilic cells (bc), cytoplasmic vacuolization (arrows), (**C**) presence of vacuole-like spaces in the tubular lumen (asterisk), (**D**) epithelial flattening with or without intraluminal eosinophilic mass (ef), and (**E**) dilated tubules (dt). (**E**) The interstitium shows large lymphocyte infiltration (L). (**F**) Kidney cortex of diabetic mice treated with WSDP. More dilated tubules (dt) and larger interstitial lymphocyte infiltrations (L) compared to diabetic mice. Bar = 20 μm.

In the kidneys of control diabetic mice treated with a physiological solution changes were observed in the renal corpuscles and in the renal tubules. Majority of Bowman’s spaces were very narrow or totally reduced (Figure 4A). Differentiation of squamous cells in the parietal layer of Bowman’s capsule into columnar cells was also noticed (Figure 4A). In one sample, Bowman’s spaces were not narrow, some were even expanded, while parietal layers of Bowman’s capsule were mostly thickened. Particular proximal tubules or their individual cells showed signs of necrosis, i.e. had more eosinophilic cytoplasm and decreased nuclei (Figure 4A-C). In most of the samples, basophilic cells with or without larger nuclei appeared in the wall of some tubules (Figure 4). Tubules with cytoplasmic vacuolization (Figure 4B-D), tubules with vacuole-like spaces in the lumen with or without granular content (Figure 4C), tubules with thinned epithelium, with or without intraluminal light eosinophilic mass (Figure 4D) and dilated tubules (Figure 4E) were also found in the outer cortex and particulary in the deep cortex and in the medulla close to deep cortex. Smaller or larger lymphocyte infiltrations were found in the cortex of all samples (Figure 4E), while in the half of them, very small infiltrations were also found in the medulla.

Kidneys of diabetic mice treated with WSDP showed similar changes to control diabetic mice but with much larger lymphocyte infiltrations and more dilated tubules in the outer cortex (Figure 4F).

Kidneys of control diabetic mice treated with an ethanolic solution had significantly less lymphocyte infiltrations and less eosinophilic proximal tubules compared to the kidneys of control diabetic mice treated with a physiological solution. Few tubules with basophilic cytoplasm were found in only one sample, while in others, basophilic tubules had light cytoplasm and larger nuclei. In one sample, tubules with thinned epithelium and eosinophilic mass in the lumen were found in greater numbers.

Kidneys of diabetic mice treated with EEP showed similar changes to control diabetic kidneys treated with an ethanolic solution. In all samples there were changes in renal corpuscles, presence of vacuolized tubules, tubules with intraluminal vacuole-like spaces, tubules with thinned epithelium with or without intraluminal eosinophilic mass and necrotic tubules. Most of the samples had few basophilic tubules, while in one sample such tubules were more numerous and occupied large area of deep cortex. In the same sample large lymphocyte infiltrations were also found. Other samples had less or more lymphocyte infiltrations compared to control. In most samples, in the outer cortex occasional tubules were more dilated compared to control. In one sample they were extremely dilated.

#### Effect of propolis preparation on lipid peroxidation of kidney and liver

**Figure 5:**
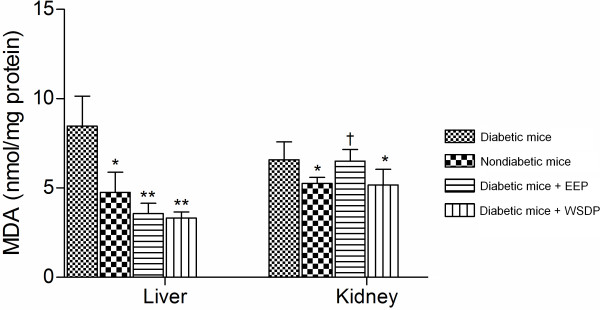
**Levels of lipid peroxidation in liver and kidney of nondiabetic mice, untreated diabetic mice and diabetic mice treated with WSDP and EEP.** Values are means ± SD (n = 4). (Diferences with untreated diabetic mice ^*^P <0.05; ^**^P <0.01; Diferences with nondiabetic mice ^†^*P* <0.05; ^‡^*P* <0.01; Student’s *t*-test)

The principle of lipid peroxidation is the reaction of MDA, an end product of lipid peroxidation, with thiobarbituric acid (TBA) to form a pink chromogen. The levels on MDA in liver and kidney of untreated diabetic animals and diabetic animals treated with WSDP and EEP are presented in Figure 5. The lipid peroxidation in liver tissue was notably improved in animals treated by EEP and WSDP (*P* <0.001). In addition, WSDP was able to lower the extent of lipid peroxidation of kindey (*P* <0.05).

### Antioxidant capacity of the extracts

**Figure 6:**
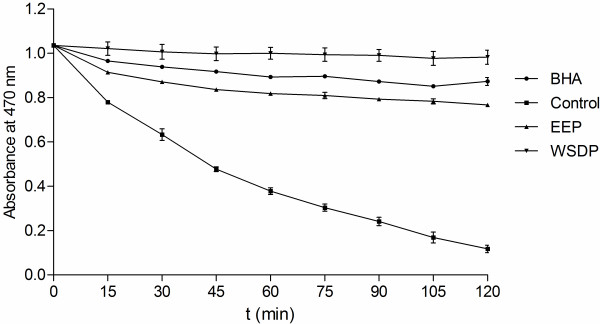
**Inhibition of bleaching of β-carotene-linoleic acid emulsion by the WSDP, EEP and BHA over 120 min.** Values are means ± SD (n = 3).

The basis of β-carotene*−*linoleic acid assay is degradation of β-carotene in reaction with linoleic acid free radical. Antioxidants present in the solution can hinder this reaction and consequently prevent discoloration of β-carotene solution.The reduction of absorbance of β-carotene*−*linoleic acid emulsion in presence of the extracts is shown in Figure 6. Comparison of the ANT values of the samples (Table 4) indicates that the WSDP was more successful at inhibition of bleaching of β-carotene emulsion than BHA (*P* <0.0001) while EEP showed lower activity than BHA (*P* <0.0001). However, the activity of both extracts was equal to the activity of BHA.

**Table 4:** **Antioxidant activity in β-carotene-linoleate test (ANT), radical scavenging activity (EC**_**50**_**), slope of reducing power trendline (SRP) and metal chelating activity (ChEC**_**50**_**) of WSDP and EEP**

	**ANT (%)**	**EC**_**50**_**(μg/ml)**	**SRP (mg**^**−1**^**)**	**ChEC**_**50**_**(μg/ml)**
EEP	87.3 ± 0.39^####^	27.82 ± 0.44^####^	0.56 ± 0.00^####^	*n.d.*
WSDP	96.81 ± 0.33^####^	55.97 ± 1.50^####^	0.53 ± 0.02^####^	47.81 ± 0.34^####^
STD	^a^91.67 ± 0.49	^a^6.02 ± 0.10	^b^7.53 ± 0.24	^c^7.18 ± 0.10

Values are means ± SD (n = 3).

*n.d*. − not detected.

STD − standard: ^a^ = BHA, ^b^ = ascorbic acid, ^c^ = EDTA.

^#^ Statistically significant difference between sample and standard (^####^*P* <0.0001; Student *t*-test).

**Figure 7:**
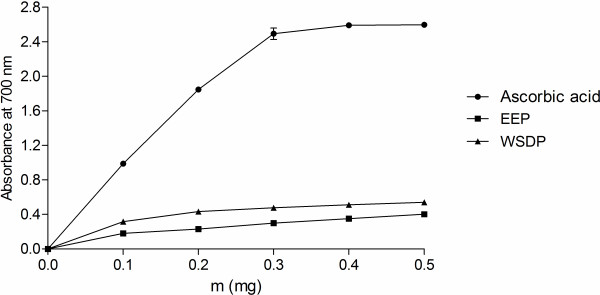
**Reducing powers of EEP, WSDP and ascorbic acid.** Values are means ± SD (n = 3).

The basis of DPPH assay is the discoloration of DPPH^·^ solution in presence of an antioxidant. In its radical form, DPPH absorbs with maximum at 517 nm, but upon reduction with an antioxidant, its absorption decreases due to the formation of its non-radical form [44]. In this study, EEP and WSDP demonstrated notable antiradical activities albeit lower than the activity of BHA (*P* <0.0001) (Table 4). In another assay performed in this study, reducing power assay, the yellow color of the test solution changes to green and blue depending on the reducing power of test specimen. Greater absorbance at 700 nm indicates greater reducing power. Figure 7 presents the reductive capabilities of the propolis extracts. Reducing power of the ascorbic acid and the extracts increased with concentration. The increase tended to be linear in case of the extracts (*r*^2^ ≥ 0.94), while the absorbance of reaction mixture with ascorbic acid remained relatively constant in higher concentrations (*r*^2^ = 0.81), most likely due to limitations of Beer–Lambert law. Thus, for purpose of comparison, slopes of the trend lines (SRP) were calculated for three lowest concentrations where *r*^2^ > 0.95 for all the samples (Table 4). The activity of WSDP and EEP was statistically equal, albeit lower than the activity of ascorbic acid (*P* <0.0001).

### Chelating activity

The chelating ability of the extracts toward ferrous ions was investigated (Table 4) in presence of ferrozin, Fe^2+^ ion chelator, which upon binding of the metal ionabsorbs with maximum at 562 nm. In the investigated concentrations (0–1000 g mL^-1^) EEP of did not show any chelating ability towards ferrous ion. WSDP, on the other hand demonstrated significant chelating ability in the present research, although lower than the ability of EDTA (*P* <0.0001).

## Discussion

In this paper the nephroprotective and hepatoprotective activities of water soluble derivative of propolis and ethanolic propolis preparation were determined in an alloxan-induced diabetic mice model. Our data showed that treatment of diabetic mice with WSDP or EEP resulted in marked increase in animal body weight (Figure 1) as well as in life span of treated mice versus untreated diabetic mice (Table 1). Interestingly, 37.5% or 50% of mice were long time survivors in untreated diabetic group versus a 100% survival of mice in diabetic group treated with WSDP or EEP. Thus, this study suggested that WSDP or EEP could be used to ameliorate oxidative stress and the adverse effects of alloxan-induced diabetes and delay the occurrence of pathogenesis of diabetic–associated diseases such as diabetic nephropathy, atherosclerosis, and brain, liver and other tissue damage in diabetes mellitus.

Body weight was rapidly reduced in animals treated with alloxan alone; the fall was the largest between 3 and 10 days, and then body weight started to recover easily. In diabetic animals treated with WSDP and/or EEP body weight was slightly reducede, especially in WSDP treated diabetic mice. Improved body weight of diabetic mice treated with WSDP or EEP could be due to a better control of hyperglycaemic state compared to the untreated diabetic mice. It is likely that decreased body weight in diabetic animals is due to dehydration and catabolism of fats and proteins [45]. Increased catabolic reactions leading to muscle wasting might also be the reason for the reduced weight gain by diabetic animals [46]. The findings of our study coincides with the findings of Fuliang et al. [47], Abo-Salem et al. [48], El-Sayed et al. [33] and Zhu et al. [31] and shows that propolis reduces total level of cholesterol and triglycerides in the blood of diabetic animals, reducing thus some of the major risk factors of cardiovascular disease. So, propolis preparations could modulate lipid metabolism (Table 3) and reduce the syndrome caused by blood lipid abnormalities [47]. Also, our data of the antioxidant effects of propolis are parallel with Abo-Salem et al. [48] who reported that both of propolis preparations, WSDP and EEP, attenuates alloxan-induced hepatotoxicity via decreasing lipid peroxidation (Figure 5). Similar results were demonstrated by Bhadauria et al. [49] who revealed that propolis may play a hepatoprotective role through reducing oxidative stress in living system. Our results show that dietary intervention with propolis preparations (WSDP, EEP) reduces free-radical-induced lipid peroxidation in liver. We could clearly observe the significant decrease (*P* <0.05) in MDA concentration after the initial 7 days of propolis supplementation. The lipid peroxidation in liver was significantly lowered in diabetic animals treated with oth EEP and WSDP in comparison with untreated diabetic mice (Figure 5). As shown in Table 3, alloxan administration induced a marked reduction in liver functions, as characterized by significant increases in serum LDH, ALP, and AST levels, in mice treated with alloxan alone compared to undiabetic mice. In addition, our results demonstrated that propolis preparations have the ability to decrease serum transferases and LDH activity in alloxan-induced diabetic animals.

On the other hand, MDA lowering effect of EEP is not observed kidney tissue. Acute alcohol consumption has been known to enhance lipid peroxidation in the cerebellum, heart and other organs [50]. Therefore, it is possible that the putative MDA lowering effects of propolis constituents were attenuated by alcohol present in the extract. These data are consistent with biochemical parameters (Table 3) indicating increased levels of urea and creatinin in the serum of untreated and propolis-treated diabetic animals in comparison with nondiabetic mice.

However, administration of propolis to diabetic mice significantly elevated hematological parameters such as the total number of red blood cells, haemoglobin and hematocrit level (RBC, and HGB, *P* <0.05; HCT, *P* <0.01 or *P <*0.05) compared to untreated diabetic mice (Table 2).

Histopathological observation of the liver sections of alloxan-induced diabetic mice showed several lesions including cellular vacuolization, cytoplasmic eosinophilia and lymphocyte infiltrations, but with individual variability. The most prominent change was the presence of smaller or larger vacuole-like spaces in the hepatocyte cytoplasm. This was probably a result of increased quantity of fat within cells due to impaired metabolism of fatty acids. The presence of single or small foci of eosinophilic cells and pale foci was another characteristic of damaged cells indicating necrosis. Similarly, Ragavan and Krishnakumari [51] found periportal vacuolization with focal necrosis in the rat liver treated with single intraperitoneal injections of alloxan in a dose of 120 mg kg^-1^ body weight, while Khalil et al. [52], with a higher dose of 150 mg kg^-1^ found disorganization of the hepatic cords and vacuolized hepatocytes with picnotic nuclei. Vinagre et al. [53] reported enlarged hepatocytes with vacuolar cytoplasm and hypertrophic nuclei, sinusoidal dilatation and lymphocyte infiltrations in the periportal regions of streptozotocin-induced diabetic rat (70 mg kg^-1^ bw). Al-Rawi [54] also showed sinusoidal dilatation, swollen hepatocytes with marked cytoplasmic vacuole, lymphocyte infiltrations around the portal veins and severe fibrosis with a lower dose of streptozotocin (50 mg kg^-1^ bw).

Our results showed that the treatment of diabetic mice with both aqueous and ethanolic extracts of propolis results in decreased number of vacuolized cells and degree of vacuolization, although ethanolic extract had stronger effect. These findings suggest that propolis treatment may be able to improve the impairment of fatty acid metabolism in diabetes.

Renal histology showed corpuscular, tubular and interstitial changes. Changes in the corpuscles were related to the reduction of Bowman’s space which could be due to the expansion of mesangial and/or endothelial cells of glomerulus. Expansion of mesangial region described Hamada and Fukagawa [55] and Teoh et al. [56] Prabhakar et al. [57] also reported extensive mesangial expansion and basement membrane thickening. The role of the mesangium is to support and anchor the capillary loops to enable them to retain their structure and function. Findings of Fioretto and Mauer [58] confirm that mesangial expansion is crucial structural change leading to a loss of renal function in diabetes. It is thought that such expansion reduces the capillary surface area available for filtration [59]. Increase in mesangium was shown to correlate with the development of proteinuria, although Wolf et al. [59] are not assured that mesangial expansion is the only cause of the proteinuria as they argue that the changes also occur within the visceral layer of Bowman’s capsule which can lead to alterations in the glomerular filtration barrier. We observed changes in the parietal layer of Bowman’s capsule characterized as metaplasia. Although parietal layer has no role in the glomerular filtration, we assume that beside glomerular expansion, the change of squamous epithelium into columnar could also contribute to the reduction of Bowman’s space. It is known that changes in the structure and function of the glomerulus affect the tubules. Our findings revealed more changes in renal tubules like dilatation, vacuolization, epithelial flattening with or without intraluminal mass and necrosis. Similar to our findings, Prabhakar et al. [57] reported tubular dilatation and atrophy, Teoh et al. [56] hypercellularity and necrosis of the proximal tubules while Baehr [60], Saundby [61], described epithelial vacuolization of the proximal tubules and the loop of Henle. We also found tubules with cytoplasmic vacuolization and tubules with intraluminal vacuole-like spaces in the deep cortex and outer medulla. Cytoplasmic vacuoles could be glycogenic or lipid as tubular cells in proteinuria reabsorbs lipids while intraluminal vacuoles could represent small drops of fatty detritus as parenchymatous degeneration is usually associated with fatty degeneration [62].

All these tubular changes point to a disturbance in their function. Our results show that treatment with an aqueous or ethanolic extract of propolis does not improve renal histopathology in diabetic mice.

Both propolis preparations, water and ethanolic extract of propolis, offer a promising therapeutic value in prevention of diabetes; propolis preparations reduce free-radical-induced lipid peroxidation in liver but it does not improve renal function in diabetic mice in period observations (9 days). Data are consistent with renal hystopathological observations. On the other hand, since all treated diabetic mice are long–time survivor, it is possible that renal repair process occurs later or that kindney damages were not lethal for animals. This thesis was confirmed by numberous authors in recent studies that have shown that dietary agents with antioxidative activity, including selenium, vitamin C, vitamin E, capsaicin from hot red peppers, and caffeic acid phenethyl ester from honeybee propolis, can attenuate drug-induced nephrotoxicity and hepatotoxicity based on ROS production [29] and [32]. It is possible that propolis may prevent hepatorenal injury by inhibiting lipid peroxidation and enhancing the activities of antioxidant enzymes as suggested by Abo-Salem et al. [48].

Propolis antioxidant activity has been demonstrated using numerous *in vitro* tests [20] and [63]. In this study, antioxidant activity was investigated using four assays which cover different aspects of antioxidant activity. Being a relatively stable free radical, DPPH^·^ is frequently used to determine radical-scavenging activity of natural compounds. DPPH assay estimates the ability of sample to scavenge free radicals, species capable of causing damage to natural macromolecules, such as nucleic acids, polysaccharides and lipids [64]. In this study, the antiradical activity of EEP was more pronounced than the activity of WSDP. This is in accordance with some previous studies which showed a superior activity of ethanolic extract over aqueous in this assay. For example, antiradical activity of ethanolic extracts of both, poplar-type propolis from Croatia and alecrim-type Brazilian propolis, was greater than the activity of their aqueous extracts [14].

In β-carotene*−*linoleic acid assay, the degradation of β-carotene occurs in reaction with linoleic acid free radical formed at elevated temperatures. Subsequent loss of conjugation leads to a decrease in absorbance at 470 nm. Antioxidants present in the solution can prevent the degradation of β-carotene by reacting with the linoleic acid free radical or any other radical formed in the solution [37]. Thus, in this assay, the capacity of antioxidants to prevent degradation of natural lipids, such as linoleic acid, is measured. The reducing power of a compound, on the other hand, is related to its electron transfer ability and may serve as a significant indicator of its potential antioxidant activity. It estimates the ability of a substance, or an extract which contains it, to donate a proton, and thus cause the transformation of free radicals into a less reactive species. Interestingly, some previous findings indicated that ethanolic propolis extracts were superior reducers and inhibitors of β-carotene degradation [14]. In this study, however, WSDP was more successful inhibitor of β-carotene bleaching than EEP, while the reducing powers of the two extracts were equal. Such differences in activity are probably caused by different method of preparation of aqueous extract used in the study of Kosalec et al. [14].

Hydroxyl radicals are among the most harmful ROS in biological systems. Free ferrous iron is sensitive to oxygen and gives rise to ferric iron and superoxide, thereby generating hydrogen peroxide. Thus formed hydrogen peroxide reacts with ferrous iron and generates the hydroxyl radical, which may subsequently oxidize surrounding biomolecules. In this process, known as the Fenton reaction, hydroxyl radical production is directly related to the concentration iron or other transition ions. In pathological states involving iron overload or impaired sequestering of iron by transport or storage proteins, Fenton chemistry is an important generator of ROS *in vivo*[65]. In the investigated concentration range (0−1 mg mL^-1^) constituents of EEP were incapable of chelating ferro ions in this assay. WSDP, on the other hand demonstrated significant chelating ability. Some previous studies showed that ethanolic extract of Brazilian propolis showed chelating abilities [66]. Brazilian propolis belongs to two groups of chemically different types of propolis, alecrim and poplar type, respectively. The differences in chemical compositions have probably caused such different behavior of Croatian and Brazilian propolis in presence of ferro ions. However, even in the study of Wang et al. [66] the activity was achieved only in relatively high concentration (EC_50_ was about 2 mg mL^-1^), which was not used in this study.

It has been reported that propolis contains various phenolic substances. Some studies demonstrated that the extracts having higher phenol content also have higher DPPH radical-scavenging activity and other types of antioxidant activities [67]. However, antioxidant activity of the WSDP was more pronounced in most of the performed assays which leads to conclusion that besides phenols some other compounds may at least partly be responsible for the antioxidant activity of investigated extracts.

## Conclusions

In conclusion, the administration of natural antioxidants such as propolis mitigate alloxan-inuduced toxicity and increase survival times of diabetic animals and attenuates hepatotoxicity and nephrotoxicity by reducing alloxan-induced oxidative stress in a diabetic mice model. The findings of the present study strongly suggest that oxidative stress has a pivotal role in the pathophysiology of diabetic nephropathy and hepatotoxicity and that propolis preparations are able to attenuate diabetic hepatorenal damage, probably through its anti-oxidative action. The study implies that propolis could be substituted as a dietary supplement to prevent or treat diabetic complications.

Therefore, supplementation with propolis preparations may be one approach that could be utilized to protect animals or patients against oxidative stress induced damage in diabetes. However, to clarify these observations further studies are needed to investigate the association between DNA damage of liver and kidney and its repair in diabetic mice treated with propolis in long-term period.

## Competing interests

There are no conflicts of interest to declare.

## Authors’ contributions

NO designed the study, performed the experiments, participated in the data acquisition, analyzed samples, gathered data, performed statistical analysis and wrote the manuscript. DS participated in the data acquisition and participated in its design and coordination. MZK analyzed and interpreted the antioxidant capacity of the test components. GG, GL analyzed and interpreted the hystopatological activity of the test components. All authors read and approved the final manuscript.

## Pre-publication history

The pre-publication history for this paper can be accessed here:

http://www.biomedcentral.com/1472-6882/12/117/prepub
